# Immediate effects of forefoot wedges on multi-segment foot kinematics during jogging in recreational runners with a symptomatic pronated foot

**DOI:** 10.3389/fphys.2022.1064240

**Published:** 2023-01-09

**Authors:** Xianyi Zhang, Benedicte Vanwanseele

**Affiliations:** ^1^ School of Biomedical Engineering, Shenzhen Campus of Sun Yat-sen University, Shenzhen, China; ^2^ Key Laboratory of Sensing Technology and Biomedical Instrument of Guangdong Province, School of Biomedical Engineering, Sun Yat-sen University, Guangzhou, China; ^3^ Department of Movement Sciences, KU Leuven, Leuven, Belgium

**Keywords:** foot orthoses, running biomechanics, multi-segment foot kinematics, pronated foot, dose-response effect

## Abstract

**Background:** Foot orthoses (FOs) have been used to alter lower limb kinematics and kinetics in pronated feet. A clear relationship between FOs’ features, e.g., the amount of wedging and support, and the corresponding biomechanical responses is vital for the design and prescription of FOs. In this study, we sought to determine if changing the level of the forefoot wedge would cause a linear response in the multi-segment foot kinematics during jogging, and if this effect would be enhanced by an arch support.

**Methods:** Ten pairs of 3D printed FOs with five levels of forefoot wedges and two levels of arch supports were tested on 12 recreational runners with a symptomatic pronated foot. Multi-segment foot kinematic data during jogging was measured using the Oxford Foot Model. Two-way ANOVAs were performed to examine the main effect of the forefoot wedge and arch support, as well as their interaction on peak joint angles. Statistical parametric mapping and paired-t tests were used to identify differences in the foot kinematic traces and the joint range of motion (ROM) between each FO and the control, respectively.

**Results:** Linear main effects for the forefoot wedge level were found in the forefoot peak dorsiflexion, eversion and rearfoot peak dorsiflexion of jogging. FOs with a medial forefoot wedge caused an average of 2.5° reduction of the forefoot peak abduction during jogging. Furthermore, forefoot wedges showed an opposite effect on the sagittal ROM of the forefoot and rearfoot. Adding an arch support did not improve the kinematic performance of a forefoot wedge during jogging.

**Conclusion:** This study highlights a linear dose-response effect of a forefoot wedge on forefoot kinematics during jogging, and suggests using a medial forefoot wedge as an anti-pronator component for controlling forefoot motion of a pronated foot.

## 1 Introduction

Foot orthoses (FOs) have been used as a conservative way to manage pain and reduce the risk of overuse injuries in individuals with a pronated foot posture (Banwell et al., 2014; Desmyttere et al., 2021). FOs designed for pronated feet target to restore their normal foot dynamic function, which can be evaluated *via* joint kinematics, kinetics and muscle activities during walking and running (Cherni et al., 2021; Hajizadeh et al., 2022). These corrective effects are usually achieved *via* appropriate configurations of orthotic components (Desmyttere et al., 2021). Thus, a clear relationship between the features of FO components and the corresponding biomechanical responses, i.e., the dose-response effect, is vital for the design and prescription of FOs.

Wedged FO components and arch supports have been used to reduce foot pronation and abnormal joint moments (Nawoczenski and Ludewig, 2004; Braga et al., 2019). Currently, the dose-response relationship between each FO component and foot biomechanics is poorly understood. Most FO studies only examine very limited variations of FOs, normally 1–2 types (Barn et al., 2014; Cherni et al., 2021; Hajizadeh et al., 2022). Only a few studies examined multiple levels of rearfoot wedges and arch supports to determine their dose-response effect on lower limb kinematics during gait. For rearfoot wedges, Telfer et al. (2013) reported a linear effect of rearfoot wedge on the peak and mean rearfoot eversions. For arch supports, Wahmkow et al. (2017) examined four different arch support heights during walking, but found there was no systematic effect on foot kinematics. Studies on the effects of forefoot wedges on kinematics were relatively limited (Nawoczenski and Ludewig, 2004).

Controlling excessive foot pronation involves reducing forefoot abduction, forefoot dorsiflexion and rearfoot eversion (Neville et al., 2016). Currently, most FOs focused on reducing the rearfoot eversion (Banwell et al., 2014; Mo et al., 2019), and consequently a majority of FO studies adopt the rearfoot wedge as the main anti-pronator FO component (Telfer et al., 2013; Cherni et al., 2021; Desmyttere et al., 2021). By comparing pronated feet with and without symptoms, previous studies suggested that the rearfoot peak eversion was comparable between two groups and thus questioned the effectiveness of reducing the rearfoot eversion to manage overuse injuries (Levinger et al., 2010; Kerr et al., 2019; Zhang et al., 2019). Alternatively, it was suggested that excessive forefoot peak abduction, which occurred during propulsion, might be associated with the injury risk in pronated feet, and that regulating forefoot transverse motion could benefit symptomatic pronated feet. The body weight transfers to the forefoot after heel-off of gait stance phase, during which the forefoot orthotic component would theoretically be more effective in altering forefoot kinematics than the rearfoot component (Hsu et al., 2014).

To improve the biomechanical performance of FOs, adding an arch support to other types of FOs has been suggested by several studies (Nakajima et al., 2009; Zhang et al., 2017). It has been shown that the additional use of an arch support improved gait stability and comfort of a heel lift FO (Zhang et al., 2017), and further reduced knee adduction moment of a laterally wedged FO (Nakajima et al., 2009). Using an arch support alone has also been shown to redistribute plantar pressure and reduces impact loading during running (Wang et al., 2020; Peng et al., 2021), while its effect on joint kinematics during running is inconsistent across literature (Wahmkow et al., 2017; Hajizadeh et al., 2020). For a forefoot wedge FO, it remains unclear if changing the arch support parameters would enhance its impact on joint kinematics or not.

Therefore, the primary aim of this study was to examine the dose-response effect of FOs with a forefoot wedge on multi-segment foot kinematics during jogging in recreational runners with a symptomatic pronated foot. We hypothesized a dose-response relationship between forefoot wedges and forefoot kinematics. The secondary aim of this study was to examine if a higher arch support would enhance the biomechanical effect of a forefoot wedge. The insights gained from this work would provide scientific evidence for foot orthoses prescription to manage excessive foot pronation and prevent running-related overuse injuries.

## 2 Methods

**FIGURE 1:**
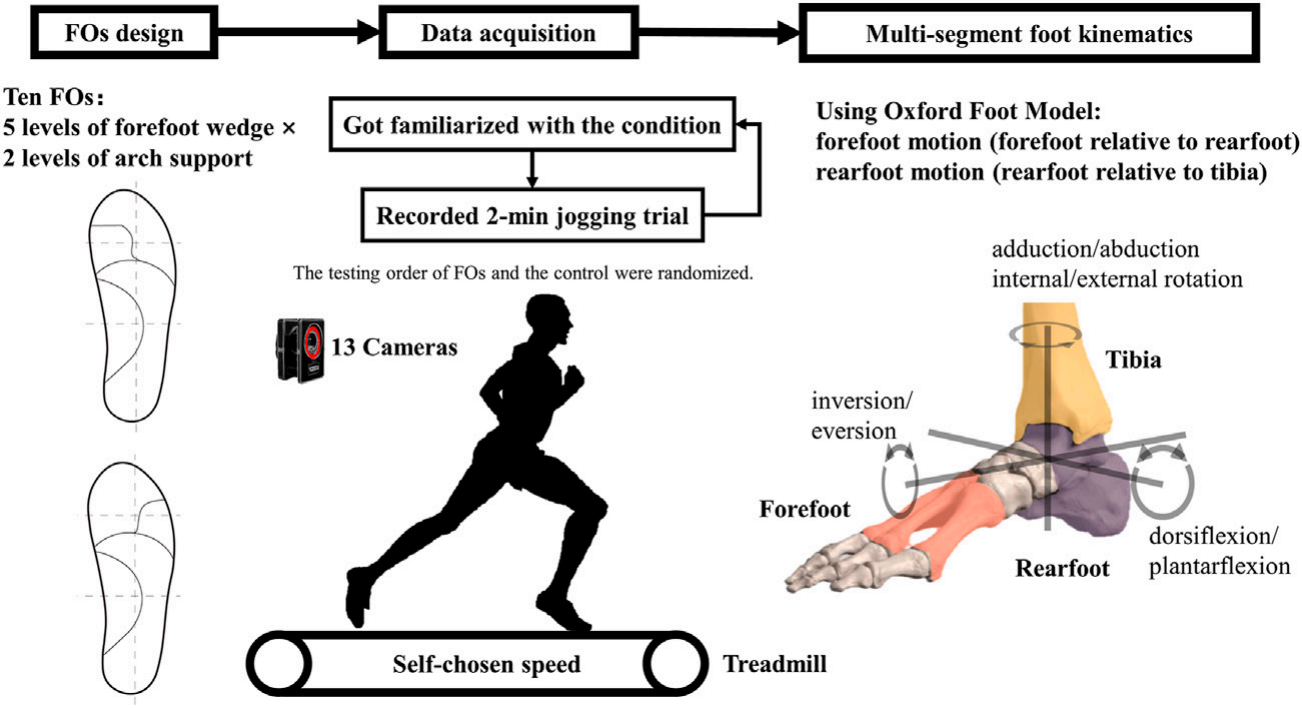
The experimental setting.

This study was approved by the Medical Ethics Committee of KU Leuven and all participants gave informed consent. The experimental setting is shown in Figure 1.

### 2.1 Participants

This study recruited 12 recreational runners (5 females and seven males) with a minimal running volume of 10 km per week. These runners also participated in different trials of our previous study, which examined the effect of FOs on the plantar pressure variables during overground running (Zhang et al., 2022a). They had an average age of 25.8 ± 5.5 years, weight of 72.5 ± 9.0 kg, height of 1.79 ± 0.08 m, and training volume of 19.9 ± 7.9 km per week. All participants were diagnosed with some form of lower-leg overuse injuries in the last 6 months before testing, including Achilles tendinopathy, plantar fasciitis, medial tibial stress syndrome, and general knee pain. They were pain free at the time of data acquisition. The most symptomatic leg, which was based on subjective report of one’s injury history, was chosen for data collection. Their foot postures were examined *via* foot posture index (FPI), with a FPI value equal or larger than six being classified as a pronated foot posture (Redmond et al., 2008). The average FPI score of all participants was 7.9 ± 1.4.

### 2.2 Foot orthoses

**FIGURE 2:**
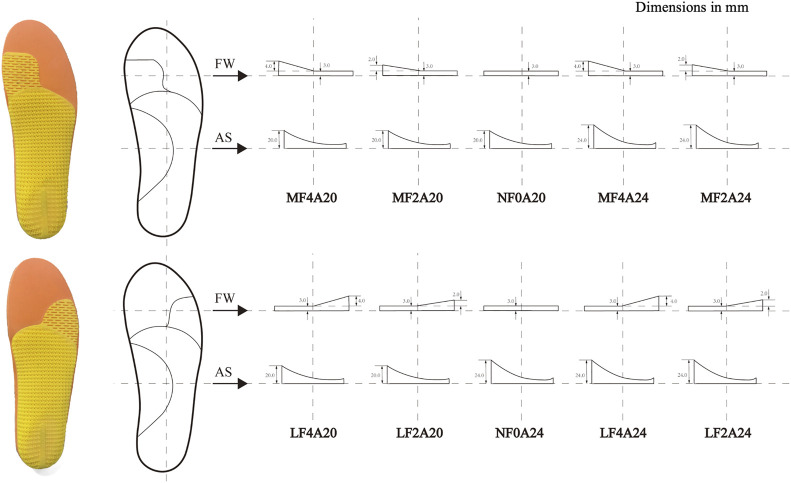
Bottom view and dimensions of ten FOs with abbreviations. FW: forefoot wedge, AS: arch support. MF4, MF2, NF0, LF2, LF4 stand for a forefoot wedge with 4 mm medial, 2 mm medial, 0 mm, 2 mm lateral, and 4 mm lateral wedge, respectively; A20 and A24 stand for an arch support with a height of 20 mm and 24 mm, respectively. The base layer in yellow was composed of a conventional three-quarter orthosis with or without a forefoot wedge. Foot orthosis was composed of a 3D printed base layer and flat full-length top layers.

The FO features were the same as described in our previous study (Zhang et al., 2022a). As shown in Figure 2, ten FOs varied in the forefoot wedges (5 levels: MF4, MF2, NF0, LF2 and LF4 with MF stands for a medial wedge, NF stands for neutral forefoot, and LF stands for a lateral wedge) and the arch supports (2 levels: A20 and A24). The abbreviations of FOs with an arch support height of 20 mm are MF4A20, MF2A20, NF0A20, LF2A20, LF4A20, and those with an arch support height of 24 mm are MF4A24, MF2A24, NF0A24, LF2A24 and LF4A24. All FOs were inserted into a standard neutral running shoe for testing.

### 2.3 Equipment and procedure

A motion capture system (Vicon MX, Vicon Motion System Ltd., Oxford, England) with 13 cameras was used at a sampling frequency of 150 Hz. Reflective markers were attached to the skin according to the lower limb Plug-in gait model (Kadaba et al., 1990) combined with the left/right Oxford Foot Model (Stebbins et al., 2006). Participants wore testing shoes (a neutral shoe model with a uniform EVA midsole) and socks with holes (within 25 mm diameter), and markers attached to the skin *via* a magnetic base through these holes. This allowed the foot marker locations to be consistent between all testing conditions. An instrumented treadmill with integrated force plates (Motekforce Link, Amsterdam, the Netherland) was used to measure the ground reaction forces (GRFs) at a sampling frequency of 900 Hz.

Before the jogging measurements, a static trial of barefoot standing was recorded for each participant. Participants jogged on the treadmill at a self-chosen speed, and this speed was kept constant in all test conditions for the same participant. For each condition, the participant walked and jogged on the treadmill for about 5 min to get familiarized with the condition tested, then a 2-min jogging trial was recorded. The average self-chosen jogging speed was 8.4 ± 0.8 km/h. The foot orthoses were used for both feet. Running trials were repeated for 11 conditions, i.e., shod only (the control) and ten FO conditions. The running strike pattern was visually checked in the Vicon Nexus software, and a rearfoot strike pattern was identified with the center of pressure at foot contact locating at the heel region. All participants adopted a rearfoot strike pattern during all trials. The testing order of FOs and the control was randomized for each participant.

### 2.4 Data analysis and statistics

Kinematic data was processed in Vicon Nexus software (Vicon MX, Vicon Motion System Ltd., Oxford, England) and low pass filtered at 15 Hz using a fourth order Butterworth filter in MATLAB (The Mathworks Inc., MA, United States). For each trial, the vertical GRF was used to detect the stance phase with a threshold of 50 N. Lower limb segments were defined as previously reported (Stebbins et al., 2006). The following kinematic variables were determined: the forefoot relative to rearfoot: dorsiflexion/plantarflexion, adduction/abduction and inversion/eversion in the sagittal, transverse and frontal planes, respectively; and the rearfoot relative to tibia: dorsiflexion/plantarflexion, internal/external rotation and inversion/eversion in the sagittal, transverse and frontal planes, respectively.

Mean kinematic values and the range of joint motion (ROM) of at least five consecutive steps of each condition of each participant were used for further statistical analysis. Two-way ANOVAs were performed to determine the main effect for the forefoot wedge, arch support, and any interaction effects. Where a significant effect was found, linear contrasts were tested to determine if this effect was linear. (Telfer et al., 2013). For this analysis, all variables were relative to the control. The data of joint ROM were normally distributed. To examine the difference in joint ROM between each FO and the control, a paired *t*-test between them was performed. To compare the time series of forefoot kinematics during the stance phase between each FO and the control, a one-dimensional statistical parametric mapping (SPM) was performed (open-source: www.spm1d.org) in Matlab. *p*-values less than 0.05 were considered statistically significant.

## 3 Results

### 3.1 Dose-response effects

**FIGURE 3:**
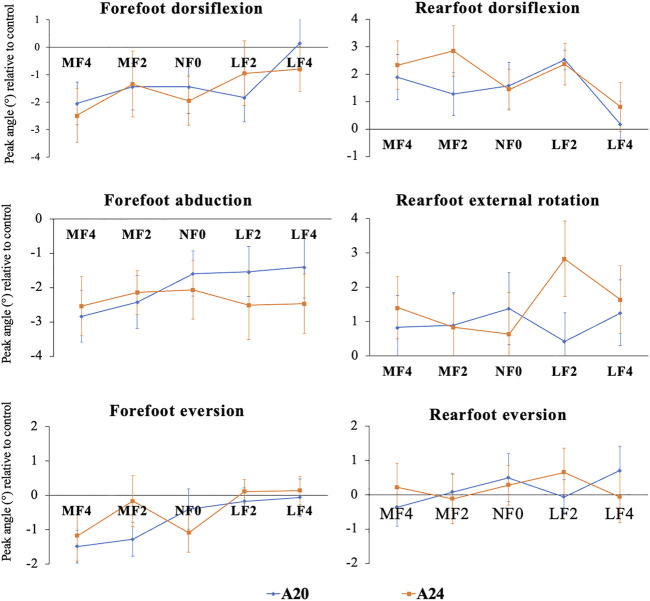
Average peak joint angles (± standard error) during the stance phase of jogging. A20 and A24: Arch support height of 20 mm and 24 mm, respectively; MF4, MF2, NF0, LF2 and LF4: forefoot component with 4 mm medial wedge, 2 mm medial wedge, none wedge, 2 mm lateral wedge and 4 mm lateral wedge, respectively. Blue line represents FOs with A20 and orange line represents FOs with A24.

Results of two-way ANOVAs are presented in Table 1. Linear main effects for the forefoot wedge level were found in the forefoot peak dorsiflexion, eversion and rearfoot peak dorsiflexion of jogging. In contrast, there was no significant main effect for the arch support level. Moreover, no interaction effects between the forefoot wedge and arch support were found. Figure 3 further illustrated the trends of peak kinematic changes by each FO in relative to the control. A larger medial forefoot wedge linearly reduced the forefoot peak dorsiflexion and eversion but increased the rearfoot peak dorsiflexion.

**TABLE 1:** Results of tests of within-subject effects from two-way ANOVAs.

Parameter	Effect	F	*p*-value	Best contrast
Forefoot peak dorsiflexion	Arch support	0.419	0.531	-
	Forefoot wedge	2.689	0.043	Linear (*p* = .001)
	Arch support * Forefoot wedge	1.099	0.369	-
Forefoot peak abduction	Arch support	3.035	0.109	-
	Forefoot wedge	1.456	0.232	-
	Arch support * Forefoot wedge	1.125	0.357	-
Forefoot peak eversion	Arch support	0.418	0.531	-
	Forefoot wedge	6.803	<.001	Linear (*p* = .001)
	Arch support * Forefoot wedge	1.406	0.248	-
Rearfoot peak dorsiflexion	Arch support	2.661	0.131	
	Forefoot wedge	7.891	<.001	Linear (*p* = .008)
	Arch support * Forefoot wedge	1.048	0.394	-
Rearfoot peak external rotation	Arch support	1.92	0.193	-
	Forefoot wedge	0.811	0.525	-
	Arch support * Forefoot wedge	1.657	0.177	-
Rearfoot peak eversion	Arch support	0.012	0.915	-
	Forefoot wedge	0.653	0.628	-
	Arch support * Forefoot wedge	1.106	0.366	-

### 3.2 Joint range of motion

Table 2 shows that the forefoot sagittal ROM was decreased by all FOs, while the rearfoot sagittal ROM was increased in comparable degrees. Most FOs reduced the forefoot transverse ROM, but the absolute change was small in value. In contrast, the transverse and frontal rearfoot ROM were not altered by FOs.

**TABLE 2:** Comparison of joint ROM (mean ± SD) between FOs and the control during the stance phase of jogging.

Conditions	ROM of forefoot motion (°)			ROM of rearfoot motion (°)		
	Sagittal plane	Transverse plane	Frontal plane	Sagittal plane	Transverse plane	Frontal plane
MF4A20	7.6 ± 1.8 *	2.8 ± 1.2 *	4.1 ± 1.4	22.5 ± 4.3 *	21.2 ± 5.9	18.3 ± 6
MF2A20	7.6 ± 2.2	2.9 ± 1.1 *	4.1 ± 1.8	22.2 ± 4.8	21.5 ± 5.4	18 ± 5.9
NF0A20	7.4 ± 1.9 *	2.7 ± 1.6 *	4.3 ± 1.8	22.5 ± 4.7 *	22.1 ± 6.2	18 ± 5.8
LF2A20	7.4 ± 2.2 *	2.6 ± 1.3 *	3.9 ± 1.7 *	22.1 ± 5.2 *	21.1 ± 5.3	18.3 ± 6
LF4A20	7.7 ± 2.5 *	2.9 ± 1.2 *	4.2 ± 1.8	21.1 ± 4	20.8 ± 5	17.6 ± 6
MF4A24	7.8 ± 1.8 *	2.7 ± 1.3 *	4.8 ± 1.5	22.6 ± 4 *	22.2 ± 4.7	19 ± 5.9 *
MF2A24	8.6 ± 3.7	3.1 ± 2.3	4.7 ± 2.4	23 ± 5.6 *	20.6 ± 5.9	17.5 ± 5.9
NF0A24	7.2 ± 1.9 *	3 ± 1.3	4.5 ± 2	22.9 ± 4.8 *	20.9 ± 5.4	17.8 ± 5.9
LF2A24	8.3 ± 2.1	2.8 ± 1.4 *	4 ± 1.8	22.8 ± 4.4 *	22.3 ± 6	18.3 ± 6.7
LF4A24	8 ± 2.1	2.5 ± 1.4 *	4.1 ± 1.7 *	21.9 ± 4.8	20.7 ± 4.8	17.2 ± 5.2
control	9.3 ± 3.2	3.6 ± 1.2	5.1 ± 2.8	20.7 ± 3.6	20.8 ± 4.2	17.1 ± 5.8

ROM: range of motion. *p < .05, vs. control.

### 3.3 Kinematic patterns

**FIGURE 4:**
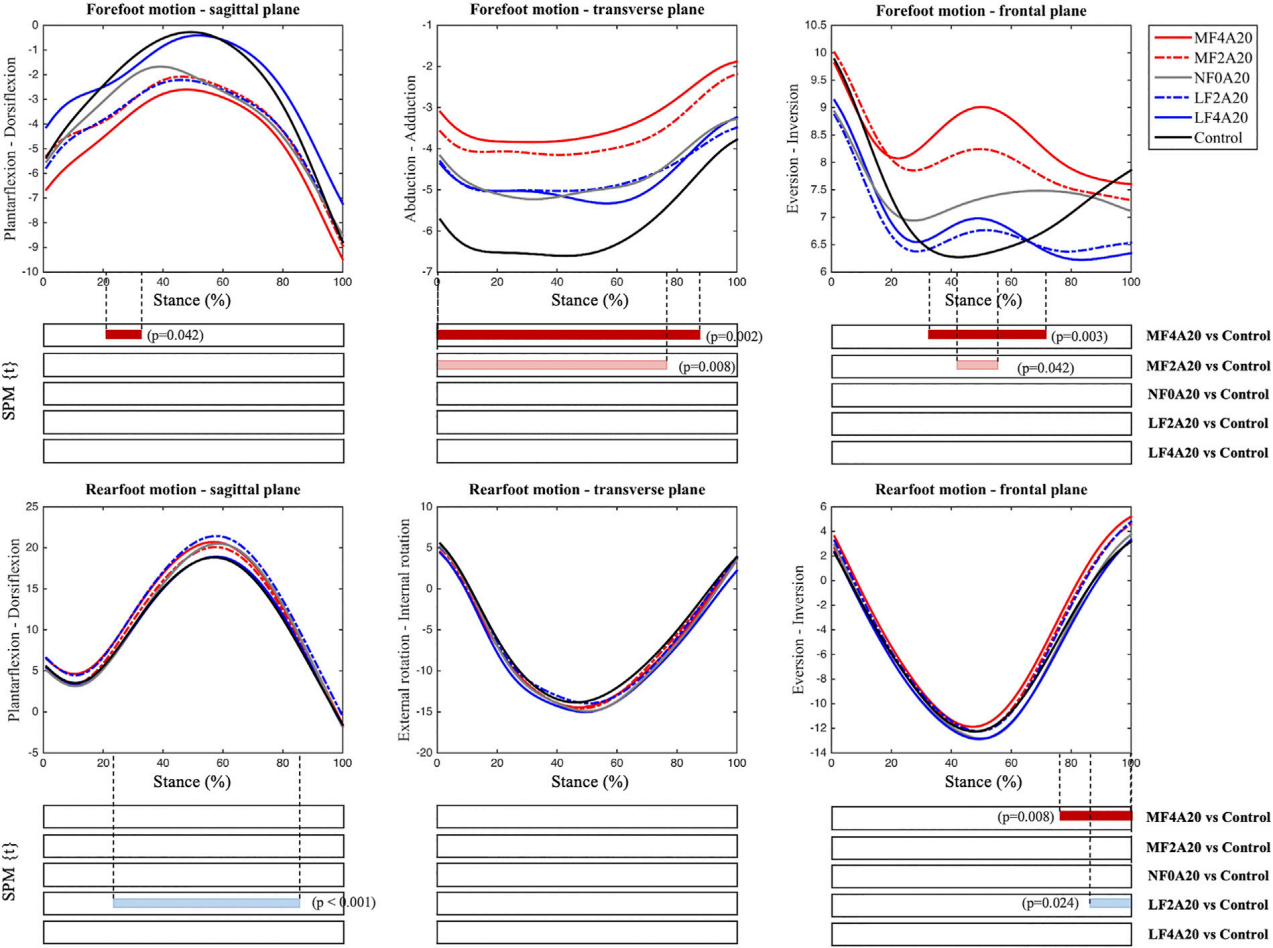
Average foot kinematic traces (°) of different FOs with an arch support height of 20 mm and the control during the stance phase of jogging. Shaded bars indicate significant differences (*p* < 0.05) between each FO and the control using SPM.

Figure 4 shows the average kinematic traces and the SPM results comparing each FO with A20 and the control. The kinematic patterns using FOs with A20 were comparable with those with A24 (see Supplementary Figure S1). Medial forefoot wedges altered the forefoot motions of three planes, reducing forefoot dorsiflexion, abduction and eversion. Compared to the control, LF2A20 significantly increased the rearfoot dorsiflexion. MF4A20 and LF2A20 reduced the rearfoot eversion during the late stance.

## 4 Discussion

The purpose of the current study was to examine the immediate effect of FOs with forefoot wedges on multi-segment foot kinematics during jogging in symptomatic pronated feet. As expected, FOs with a forefoot wedge had a significant influence on the forefoot motion, with a medial wedge reducing forefoot peak dorsiflexion, abduction and eversion during jogging. In contrary to our hypothesis, changing the arch support height neither enhanced nor weakened the impact of forefoot wedges on foot kinematics. These findings may provide insights for FOs mechanisms and references for future FOs design and prescription.

Similar to the relationship between rearfoot wedges and rearfoot eversion (Telfer et al., 2013), our results found a linear dose-response effect of forefoot wedges on forefoot eversion during jogging. Less forefoot eversion with a larger medial forefoot wedge agreed with the findings of a previous study on walking (Hsu et al., 2014), and it may also help explain why the COP was shifted more laterally with a medial forefoot wedge (Zhang et al., 2022a). Furthermore, a greater level of a medial forefoot wedge decreased the forefoot peak dorsiflexion but increased the rearfoot peak dorsiflexion, indicating a compensatory motion control mechanism of FOs on different segments of the lower limb kinetic chain. This mechanism was further confirmed by the opposite effects of current FOs on the forefoot and rearfoot ROM in the sagittal plane (Table 2). These findings are aligned with the Howard Dananberg’s Sagittal Plane Facilitation Theory (Dananberg, 1986), which suggests that restriction of sagittal plane motion at one site requires compensation by another in the chain. These kinematic alterations in a pronated foot of this study were closer to normal kinematic features of a healthy neutral foot (Hosl et al., 2014). Findings on these dose-response effects of FOs could provide reference values for FO components’ parameters to reach desired kinematic alterations, contributing to better FO customization to satisfy individual biomechanical needs.

As aforementioned, reducing the forefoot peak abduction may improve clinical outcomes for symptomatic pronated feet (Levinger et al., 2010; Kerr et al., 2019; Zhang et al., 2019). However, there is no consensus on which FO component can effectively control forefoot abduction (Barn et al., 2014; Garbalosa et al., 2015). Changing FO stiffness with a carbon fiber plate had little effect on forefoot kinematics (Rao et al., 2010; Desmyttere et al., 2021). Both medial and lateral rearfoot wedges failed to reduce the forefoot peak abduction (Telfer et al., 2013). The current study showed that a medial forefoot wedge caused an average reduction of 2.5 on the forefoot peak abduction. The amount of reduction was comparable to other joint angular changes caused by FOs (Desmyttere et al., 2018; Simonsen et al., 2021). Although the clinical significance of this reduction is unclear, it is more than 70% of the mean difference in the forefoot peak abduction between a symptomatic and asymptomatic pronated foot (Zhang et al., 2022b). Future prospective studies are required to investigate the potential clinical effect of this kinematic alteration on reducing complaints in a pronated foot population.

In agreement with our results, a recent study, which used an artificial neural network to predict the deformation of the FO, found that forefoot abduction affected the deformation in the forefoot part of the FO after heel-off of walking in flatfeet (Hajizadeh et al., 2022). However, systematically altering the forefoot peak abduction by changing the level of a forefoot wedge was not supported by the current study. This is not surprising, as the inclination of a medial/lateral forefoot wedge lies in the frontal plane, which only indirectly can manipulate foot motion in the transverse plane motion. An orthotic component in the transverse plane has been adopted in the ankle-foot orthoses to reduce forefoot abduction (Neville et al., 2016). But such component is difficult to be fitted in conventional footwear, which limits its application to FOs. Alternatively, future investigations may implement such modifications in the design of shoe last, which determines the inner space of a shoe, to systematically alter foot motions in the transverse plane.

In contrary to the positive biomechanical effects of adding an arch support to other types of FOs (Nakajima et al., 2009; Zhang et al., 2017), there was no interaction effect between an arch support and a forefoot wedge in this study. It should be noted that former studies evaluated the kinetic responses to FOs, such as the plantar pressure and knee adduction moment, while this study measured multi-segment foot kinematics. Theoretically, the arch support functions to plantarflex the first ray to increase 1st metatarsophalangeal joint (MTPJ), while the medial forefoot wedge functions to dorsiflex the 1st MTPJ. As such, this result is not surprising. Furthermore, using an arch support to modulate foot kinematics in a desirable manner is challenging. By examining prefabricated arch supports of various height, Wahmkow et al. (2017) also did not find a clear systematic effect of the arch support height on foot kinematics. The features of the longitudinal arch (LA), including its static architecture and dynamic deformation of gait, may play an important role in one’s biomechanical responses to an arch support. The LA is passively modulated by ligamentous structures and actively modulated by intrinsic and extrinsic foot muscles (Farris et al., 2020). As such, individual characteristics in foot structures may provide additional information to help understand the motion control mechanisms of an arch support in the future.

The results from this study provide further evidence to support the use of FOs in controlling foot pronation during jogging. There were, however, some limitations that should be considered. Firstly, the foot kinematic model relied on skin-mounted markers and was thus susceptible to skin movement artefact. Secondly, a treadmill was used to ensure that each participant ran at a constant jogging speed across all conditions, but most participants habitually ran over ground. Thirdly, the design of FOs in the current study did not consider the 3D foot plantar shape of each participant. Fourthly, this study was conducted on a small sample size and did not consider the gender effects on the results. It should also be noted that the symptoms of participants may not be necessarily due to biomechanical features of a pronated foot. Further prospective studies on a larger sample size is required. Finally, in order to test all FOs in 1 day without fatiguing the participants, they were only given a few minutes to warm up with each FO before testing until they felt comfortable with the testing FO. The FO testing order was randomized, and the observed running strike pattern was consistent across all conditions. Nevertheless, kinematic adaptations may occur with a long-time use of FOs, as it has been documented that muscular adaptations occurred after an 8-week intervention of FOs (Jung et al., 2011).

## 5 Conclusion

Our results suggested a linear dose-response effect of forefoot wedges on the forefoot peak dorsiflexion, peak eversion and rearfoot peak dorsiflexion during jogging in symptomatic individuals with a pronated foot posture, providing a reference for future FO design and prescription. Regarding foot pronation control, a medial forefoot wedge could be used as an anti-pronator component for restraining forefoot abduction during jogging, with 29%–43% reduction on the peak forefoot abduction. Whether this reduction is of clinical significance still requires further investigation. Furthermore, adding an arch support did not alter the kinematic performance of forefoot wedges, and future studies should consider individual differences in the LA features. This study highlights the multi-segment foot kinematic responses to different configurations of FO components. Understanding these dose-response relationships can further allow a more personalized FO design to better improve lower limb biomechanics for individuals with abnormal foot postures.

Practical implications:

1) FO with a medial forefoot wedge could reduce the peak forefoot abduction in symptomatic pronated feet during jogging.
2) This study found a linear dose-response effect of a forefoot wedge on the forefoot peak dorsiflexion, eversion and rearfoot peak dorsiflexion of jogging.
3) Adding an arch support did not alter the effects of a forefoot wedge on multi-segment foot kinematics.

## Data Availability

The original contributions presented in the study are included in the article/Supplementary Material, further inquiries can be directed to the corresponding author.

## References

[B1] Banwell H. A. Mackintosh S. Thewlis D. (2014). Foot orthoses for adults with flexible pes planus: A systematic review. J. Foot Ankle Res. 7, 23. 10.1186/1757-1146-7-23 24708560 PMC4108129

[B2] Barn R. Brandon M. Rafferty D. Sturrock R. D. Steultjens M. Turner D. E. (2014). Kinematic, kinetic and electromyographic response to customized foot orthoses in patients with tibialis posterior tenosynovitis, pes plano valgus and rheumatoid arthritis. Rheumatol. Oxf. 53, 123–130. 10.1093/rheumatology/ket337 PMC3862974 24097135

[B3] Braga U. M. Mendonça L. D. Mascarenhas R. O. Alves C. O. A. Filho R. G. T. Resende R. A. (2019). Effects of medially wedged insoles on the biomechanics of the lower limbs of runners with excessive foot pronation and foot varus alignment. Gait Posture 74, 242–249. 10.1016/j.gaitpost.2019.09.023 31574408

[B4] Cherni Y. Desmyttere G. Hajizadeh M. Bleau J. Mercier C. Begon M. (2021). Effect of 3D printed foot orthoses stiffness on muscle activity and plantar pressures in individuals with flexible flatfeet: A statistical non-parametric mapping study. Clin. Biomech. (Bristol, Avon) 92, 105553. 10.1016/j.clinbiomech.2021.105553 34973589

[B5] Dananberg H. J. (1986). Functional hallux limitus and its relationship to gait efficiency. J. Am. Podiatr. Med. Assoc. 76, 648–652. 10.7547/87507315-76-11-648 3814239

[B6] Desmyttere G. Hajizadeh M. Bleau J. Begon M. (2018). Effect of foot orthosis design on lower limb joint kinematics and kinetics during walking in flexible pes planovalgus: A systematic review and meta-analysis. Clin. Biomech. (Bristol, Avon) 59, 117–129. 10.1016/j.clinbiomech.2018.09.018 30227277

[B7] Desmyttere G. Hajizadeh M. Bleau J. Leteneur S. Begon M. (2021). Anti-pronator components are essential to effectively alter lower-limb kinematics and kinetics in individuals with flexible flatfeet. Clin. Biomech. (Bristol, Avon) 86, 105390. 10.1016/j.clinbiomech.2021.105390 34044295

[B8] Farris D. J. Birch J. Kelly L. (2020). Foot stiffening during the push-off phase of human walking is linked to active muscle contraction, and not the windlass mechanism. J. R. Soc. Interface 17, 20200208. 10.1098/rsif.2020.0208 32674708 PMC7423437

[B9] Garbalosa J. C. Elliott B. Feinn R. Wedge R. (2015). The effect of orthotics on intersegmental foot kinematics and the EMG activity of select lower leg muscles. Foot (Edinb) 25, 206–214. 10.1016/j.foot.2015.07.005 26362236

[B10] Hajizadeh M. Desmyttere G. Carmona J. P. Bleau J. Begon M. (2020). Can foot orthoses impose different gait features based on geometrical design in healthy subjects? A systematic review and meta-analysis. Foot (Edinb) 42, 101646. 10.1016/j.foot.2019.10.001 32045719

[B11] Hajizadeh M. Desmyttere G. Ménard A. L. Bleau J. Begon M. (2022). Understanding the role of foot biomechanics on regional foot orthosis deformation in flatfoot individuals during walking. Gait Posture 91, 117–125. 10.1016/j.gaitpost.2021.10.015 34673447

[B12] Hosl M. Bohm H. Multerer C. Doderlein L. (2014). Does excessive flatfoot deformity affect function? A comparison between symptomatic and asymptomatic flatfeet using the Oxford foot model. Gait Posture 39, 23–28. 10.1016/j.gaitpost.2013.05.017 23796513

[B13] Hsu W. H. Lewis C. L. Monaghan G. M. Saltzman E. Hamill J. Holt K. G. (2014). Orthoses posted in both the forefoot and rearfoot reduce moments and angular impulses on lower extremity joints during walking. J. Biomech. 47, 2618–2625. 10.1016/j.jbiomech.2014.05.021 24968944

[B14] Jung D. Y. Koh E. K. Kwon O. Y. (2011). Effect of foot orthoses and short-foot exercise on the cross-sectional area of the abductor hallucis muscle in subjects with pes planus: A randomized controlled trial. J. Back Musculoskelet. Rehabil. 24, 225–231. 10.3233/BMR-2011-0299 22142711

[B15] Kadaba M. P. Ramakrishnan H. K. Wootten M. E. (1990). Measurement of lower extremity kinematics during level walking. J. Orthop. Res. 8, 383–392. 10.1002/jor.1100080310 2324857

[B16] Kerr C. M. Zavatsky A. B. Theologis T. Stebbins J. (2019). Kinematic differences between neutral and flat feet with and without symptoms as measured by the Oxford foot model. Gait Posture 67, 213–218. 10.1016/j.gaitpost.2018.10.015 30368208

[B17] Levinger P. Murley G. S. Barton C. J. Cotchett M. P. Mcsweeney S. R. Menz H. B. (2010). A comparison of foot kinematics in people with normal- and flat-arched feet using the Oxford Foot Model. Gait Posture 32, 519–523. 10.1016/j.gaitpost.2010.07.013 20696579

[B18] Mo S. Leung S. H. S. Chan Z. Y. S. Sze L. K. Y. Mok K. M. Yung P. S. H. (2019). The biomechanical difference between running with traditional and 3D printed orthoses. J. Sports Sci. 37, 2191–2197. 10.1080/02640414.2019.1626069 31156031

[B19] Nakajima K. Kakihana W. Nakagawa T. Mitomi H. Hikita A. Suzuki R. (2009). Addition of an arch support improves the biomechanical effect of a laterally wedged insole. Gait Posture 29, 208–213. 10.1016/j.gaitpost.2008.08.007 18824355

[B20] Nawoczenski D. A. Ludewig P. M. (2004). The effect of forefoot and arch posting orthotic designs on first metatarsophalangeal joint kinematics during gait. J. Orthop. Sports Phys. Ther. 34, 317–327. 10.2519/jospt.2004.34.6.317 15233393

[B21] Neville C. Bucklin M. Ordway N. Lemley F. (2016). An ankle-foot orthosis with a lateral extension reduces forefoot abduction in subjects with stage II posterior tibial tendon dysfunction. J. Orthop. Sports Phys. Ther. 46, 26–33. 10.2519/jospt.2016.5618 26654572 PMC5771476

[B22] Peng Y. Wong D. W. Chen T. L. Wang Y. Zhang G. Yan F. (2021). Influence of arch support heights on the internal foot mechanics of flatfoot during walking: A muscle-driven finite element analysis. Comput. Biol. Med. 132, 104355. 10.1016/j.compbiomed.2021.104355 33812264

[B23] Rao S. Baumhauer J. F. Tome J. Nawoczenski D. A. (2010). Orthoses alter *in vivo* segmental foot kinematics during walking in patients with midfoot arthritis. Arch. Phys. Med. Rehabil. 91, 608–614. 10.1016/j.apmr.2009.11.027 20382295

[B24] Redmond A. C. Crane Y. Z. Menz H. B. (2008). Normative values for the foot posture index. J. Foot Ankle Res. 1, 6. 10.1186/1757-1146-1-6 18822155 PMC2553778

[B25] Simonsen M. B. Hirata R. P. Næsborg-Andersen K. Leutscher P. D. C. Hørslev-Petersen K. Woodburn J. (2021). Different types of foot orthoses effect on gait mechanics in patients with rheumatoid arthritis. J. Biomech. 139, 110496. 10.1016/j.jbiomech.2021.110496 33994179

[B26] Stebbins J. Harrington M. Thompson N. Zavatsky A. Theologis T. (2006). Repeatability of a model for measuring multi-segment foot kinematics in children. Gait Posture 23, 401–410. 10.1016/j.gaitpost.2005.03.002 15914005

[B27] Telfer S. Abbott M. Steultjens M. P. Woodburn J. (2013). Dose-response effects of customised foot orthoses on lower limb kinematics and kinetics in pronated foot type. J. Biomech. 46, 1489–1495. 10.1016/j.jbiomech.2013.03.036 23631857

[B28] Wahmkow G. Cassel M. Mayer F. Baur H. (2017). Effects of different medial arch support heights on rearfoot kinematics. PLoS One 12, e0172334. 10.1371/journal.pone.0172334 28257426 PMC5336196

[B29] Wang Y. Lam W. K. Wong C. K. Park L. Y. Tan M. F. Leung A. K. L. (2020). Effectiveness and reliability of foot orthoses on impact loading and lower limb kinematics when running at preferred and nonpreferred speeds. J. Appl. Biomech. 37, 66–73. 10.1123/jab.2019-0281 33232937

[B30] Zhang X. Li B. Hu K. Wan Q. Ding Y. Vanwanseele B. (2017). Adding an arch support to a heel lift improves stability and comfort during gait. Gait Posture 58, 94–97. 10.1016/j.gaitpost.2017.07.110 28763715

[B31] Zhang X. Pauel R. Deschamps K. Jonkers I. Vanwanseele B. (2019). Differences in foot muscle morphology and foot kinematics between symptomatic and asymptomatic pronated feet. Scand. J. Med. Sci. Sports 29, 1766–1773. 10.1111/sms.13512 31278774

[B32] Zhang X. Lam W. K. Vanwanseele B. (2022a). Dose-response effects of forefoot and arch orthotic components on the center of pressure trajectory during running in pronated feet. Gait Posture 92, 212–217. 10.1016/j.gaitpost.2021.11.033 34864487

[B33] Zhang X. Yang F. Zhao K. Vanwanseele B. (2022b). Symptomatic and asymptomatic pronated feet show differences in the forefoot abduction motion during jogging, but not in the arch deformation. Sports Biomech., 1–12. 10.1080/14763141.2022.2109506 35959794

