# Supplementary material for: Immediate effects of forefoot wedges on multi-segment foot kinematics during jogging in recreational runners with a symptomatic pronated foot
Source: Front Physiol. 2023 Jan 9;13:1064240. doi: 10.3389/fphys.2022.1064240 (PMC9868581; doi:10.3389/fphys.2022.1064240)
Supplement: Supplementary file 1 [file Image1.pdf]

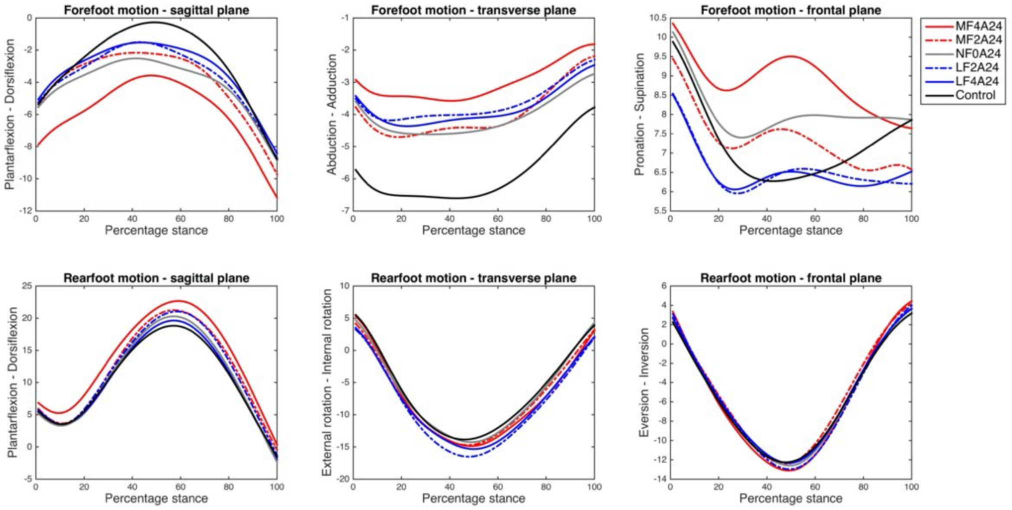

Figure S1 Average foot kinematic traces (°) of different FOs with an arch support height of 24 mm and the control during the stance phase of jogging.
